# A five year descriptive analysis of potentially preventable hospitalisations for Ear, Nose, and Throat conditions in regional Victoria, Australia, from 2015 to 2020

**DOI:** 10.1186/s12889-023-16448-3

**Published:** 2023-08-12

**Authors:** Susan O’Neill, Stephen Begg, Evelien Spelten

**Affiliations:** Department of Community and Allied Health, La Trobe University, La Trobe Rural Health School, Bendigo, VIC 3550 Australia

**Keywords:** Ear, Nose, Throat, ENT, Descriptive analysis, Potentially preventable hospitalisations, PPH, Quantitative study

## Abstract

**Background:**

Potentially preventable hospitalisations of ear, nose, and throat conditions in the Murray Primary Health Network region have been found to be higher than the state average of Victoria, Australia. This study aimed to examine the association between selected patient-level characteristics and the likelihood of residing in a Murray PHN postcode with higher than expected numbers of potentially preventable ENT hospitalisations.

**Methods:**

Unit record hospital separation data were obtained from the Victorian Admitted Episodes Dataset. Postcodes were classified as having higher than expected numbers of potentially preventable hospitalisations across three subgroups of ENT using indirect standardisation techniques. Differences between patients from ‘higher than expected’ postcodes and ‘other’ postcodes with respect to the distribution of demographic and other patient characteristics were determined using chi-squared tests for each ENT subgroup. The results were confirmed by logistic regression analyses using resident of a postcode with higher than expected hospitalisations as the outcome variable.

**Results:**

Of the 169 postcodes located in the catchment area, 15 were identified as having higher than expected numbers of upper respiratory tract infection hospitalisations, 14 were identified for acute tonsillitis, and 12 were identified for otitis media. Patients from postcodes with ‘higher than expected’ hospitalisations for these conditions were more likely than others to be aged between 0 and 9 years, Indigenous, or from a culturally and linguistically diverse background.

**Conclusion:**

Further investigation of the identified postcodes is warranted to determine access to and utilisation of primary healthcare services in the management of PPH ENT conditions in the region.

**Supplementary Information:**

The online version contains supplementary material available at 10.1186/s12889-023-16448-3.

## Background

The Perils of Place report by the Grattan Institute identified four locations in regional Victoria, Australia, as hotspots for Ear, Nose, and Throat (ENT) conditions [1], in that they have recorded rates of potentially preventable hospitalisations (PPH) for ENT conditions at least fifty percent higher than the state average in every year for a decade [1]. Two of these locations are based in the Murray Primary Health Network (Murray PHN) region.

PPH are conditions for which hospitalisation is considered to be avoidable through preventive care and early disease management, usually delivered in the primary health care setting [2–4]. PPH place unnecessary burden on emergency departments and exacerbate public health care costs [5, 6]. From 2016‐2017, there were 715 000 PPH admissions in Australia, representing approximately 8.4% of all public hospital inpatient activity [7]. PPH are an indication of access to and effectiveness of primary health care services and are of interest to health service providers and public health research, as they characterise the incidence of avoidable morbidity, providing an evidence-based understanding from which targeted interventions can be developed [2, 8].

ENT-related problems comprise of up to one quarter of adult consultations in the primary care setting, and half of paediatric consultations [9]. Additionally, ENT presentations account for one quarter of first recorded diagnosis in emergency departments [9]. There are five broad ENT categories for which hospitalisation is considered to be potentially preventable. These are classified in the Australia’s National Healthcare Agreement and include suppurative and unspecified otitis media, acute pharyngitis, acute tonsillitis, acute upper respiratory infections of multiple and unspecified sites, and chronic pharyngitis [2]. These conditions are considered to be manageable through timely treatment in primary care [2].

The Perils of Place report recommends place-based interventions to target hotspots, warranting further investigation within the Murray PHN region to understand the PPH ENT conditions, and at-risk population groups. This study aimed to examine the association between patient characteristics and PPH for ENT conditions from 2015 to 2020 in the Murray PHN region. More specifically, this paper aims to identify the clinically meaningful ENT subgroups of PPH, map the identified postcodes of significance for potentially preventable ENT hospitalisations, and identify the compounding selected patient characteristics of the PPH for ENT conditions.

## Methods

### Data sources

Unit record hospital separation data were obtained from the Victorian Admitted Episodes Dataset (VAED) for the calendar years of 2015 to 2020. This dataset was created and provided by the Victorian Agency of Health Information (VAHI). The VAED is a minimum dataset containing demographic, clinical and administrative data for all admitted episodes of care occurring in every Victorian acute hospital (both public and private) [10]. Clinical data are stored as ICD-10-AM codes in 40 diagnosis and procedure fields. Records were selected if the primary diagnosis field indicated an ENT condition and the postcode field indicated the patient’s usual place of residence was within the Murray PHN catchment. The relevant ICD-10-AM codes for ENT conditions were J02, J03, J06, J312 and H66, as defined by the National Healthcare Agreement: PI 18–Selected PPH, 2021 [2]. The postcodes that comprise the Murray PHN catchment were obtained from the Australian Bureau of Statistics (ABS) [11].

Individual ICD-10-AM codes that comprise the ENT PPH definition used in the National Healthcare Agreement were categorised into clinically meaningful subgroups on the basis of clinical information in the description that accompanies each code, as follows: acute suppurative otitis media, other chronic suppurative otitis media, suppurative otitis media unspecified, and otitis media unspecified were grouped as otitis media; streptococcal pharyngitis, acute pharyngitis dt oth spec organisms, acute pharyngitis unspecified, acute laryngopharyngitis, other acute URTI of multiple sites, acute URTI unspecified, and chronic pharyngitis were grouped as upper respiratory tract infections; and streptococcal tonsillitis, acute tonsillitis dt oth spec organisms, and acute tonsillitis unspecified were grouped as acute tonsillitis. Each postcode in the Murray PHN was classified as having a significantly higher than expected number of hospital presentations or not for each ENT subgroup using the indirect standardisation technique (Additional file 2). Estimated resident population figures for each postcode by age group (0–4, 5–9,10–14…85 +) for the year 2020 were obtained from the Australian Bureau of Statistics [11]. Age-specific rates for Murray PHN were used as the standard and Chi-squared based approximation techniques were used to calculate 95% confidence intervals (CIs). A postcode with a lower CI of greater than one was considered to have a hospitalisation rate ‘higher than expected’ compared to the average for the Murray PHN.

### Variables

Each hospital record was classified according to whether the presenting patient resided in a postcode with ‘higher than expected’ hospitalisations for the corresponding ENT subgroup, as defined above. Other variables available for analysis at the individual level were age (grouped into three categories, 0–9, 10–19, and 20 + years old), sex and Indigenous status of the patient, whether the patient required an interpreter, whether the patient mainly spoke a language other than English (LOTE) at home, whether the patient was presenting for emergency or elective reasons, whether the patient was admitted as a public or private patient, and whether the patient was subsequently transferred to another hospital at the conclusion of that care episode.

### Statistical analysis

Differences between patients from ‘higher than expected’ postcodes and ‘other’ postcodes with respect to the distribution of demographic and other patient-level characteristics were determined using chi-squared tests for each ENT subgroup. The results were confirmed by logistic regression analyses using resident of a postcode with higher than expected hospitalisations as the outcome variable. Two approaches were explored for each ENT subgroup. The first examined the bivariate relationship between resident of a ‘higher than expected’ postcode and each patient characteristic separately. The second examined the multivariate relationship between resident of a ‘higher than expected’ postcode and all patient characteristics simultaneously. Analyses were completed using a combination of Microsoft Excel and Stata 17.

Ethics approval was granted by the La Trobe University Human Ethics Committee (ethics approval number HEC19064). The institutional review board of La Trobe University (La Trobe University Human Ethics Committee—certified code EC00226) provided a waiver of informed consent. All methods were carried out in accordance with the Declaration of Helsinki.

## Results

### Clinically meaningful subgroups of ENT PPHs

There were 4816 hospital separations in the Murray PHN between 2015 and 2020 with a primary diagnosis of ENT as defined by the PPH framework in the National Health Agreement. These could be classified into three clinically meaningful subgroups as follows: upper respiratory tract infections (URTI) (2328 separations, or 48.3%), acute tonsillitis (AT) (1832 separations, or 38.0%) and otitis media (OM) (656 separations, or 13.6%). Within each subgroup, the ‘unspecified’ ICD10-AM code for that subgroup was by far the most common code used (83.7%, 91.3% and 78.7%, respectively), preventing a more detailed classification of ENT hospitalisations into subgroups. Additional file 1 provides further details.

### Postcodes of potentially preventable ENT hospitalisations

**Fig. 1:**
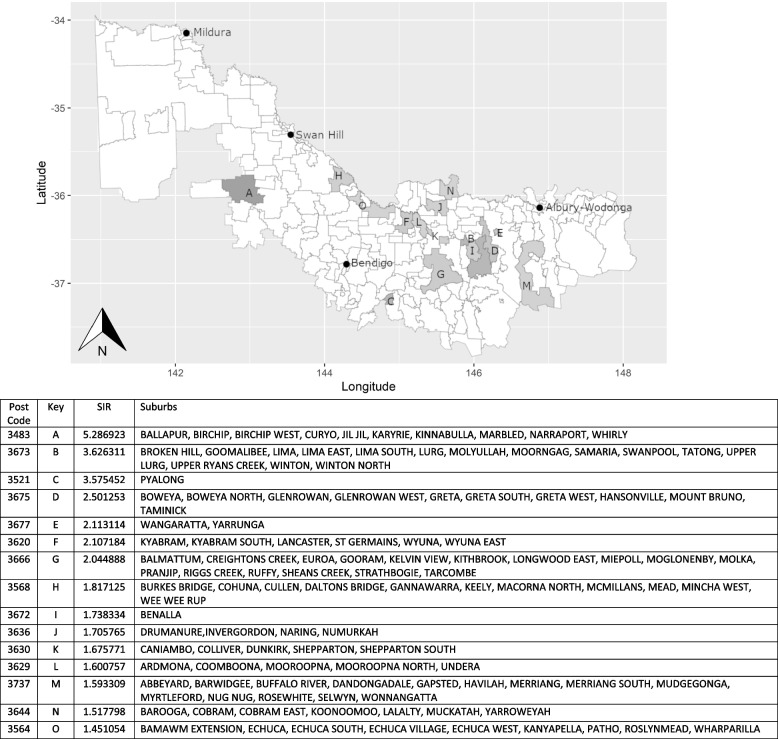
Upper respiratory tract infection

**Fig. 2:**
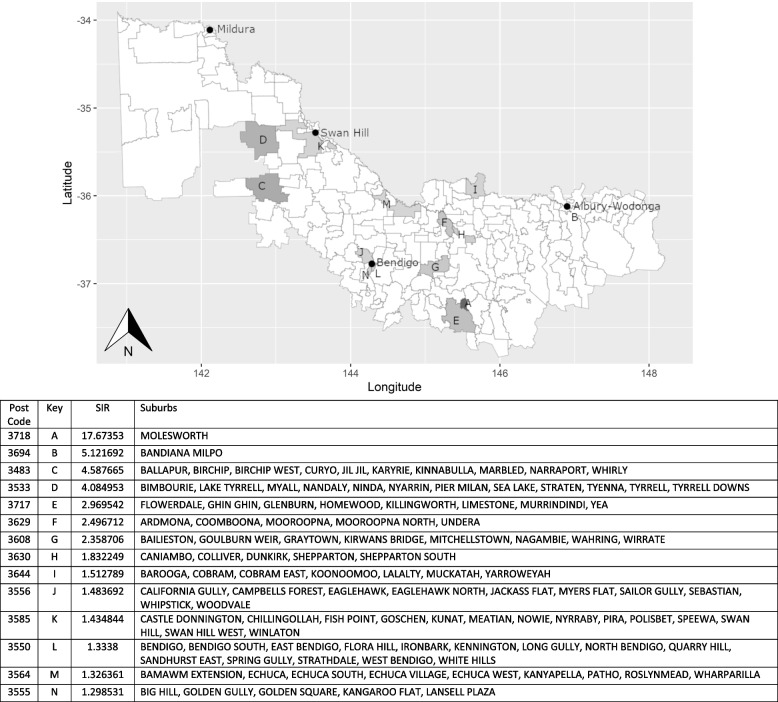
Acute tonsillitis

**Fig. 3:**
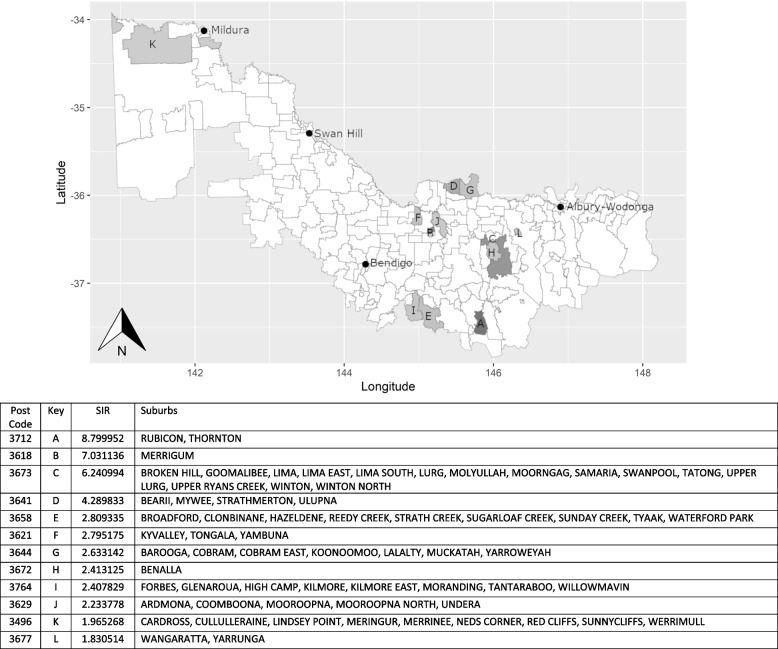
Otitis media

Of the 169 postcodes located within the Murray PHN catchment, 15 were identified as having higher than expected numbers of URTI hospitalisations, 14 were identified as having higher than expected numbers of AT hospitalisations and 12 were identified as having higher than expected numbers of OM hospitalisations. The relevant postcodes and the associated suburbs are depicted on Figs. 1, 2, and 3, respectively. The regional cities are named on the maps to provide context as to the towns distance from major health care resources.

**Fig. 4:**
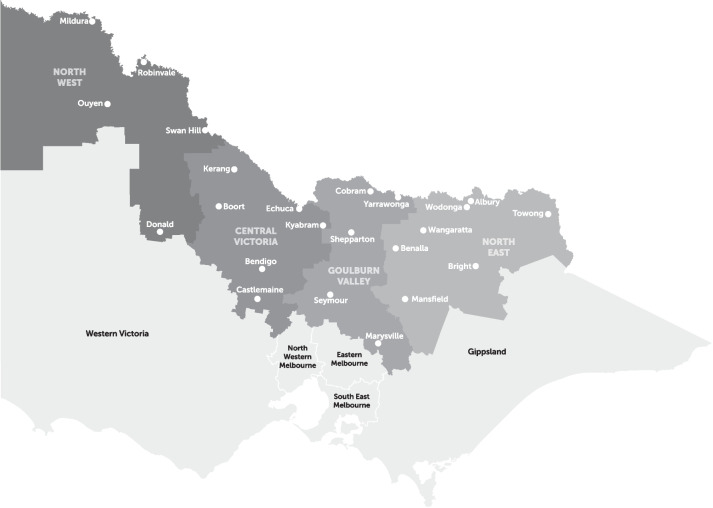
Murray PHN subregions of Victoria

The Murray PHN has four subregions: the North West, Central VIC, Goulburn Valley, and North East (see Fig. 4). Two postcodes overlapped in all three ENT subgroups, namely Ardmona (3629) and Koonoomoo (3644) (bordering with NSW), both of which are located in the Goulburn Valley. OM and URTI overlapped in three postcodes, namely Upper Ryans Creek (3673), Benalla (3672), and Wangaratta (3677), all located in the North East region. URTI and AT also overlapped in three postcodes, namely Narraport (3483) (located in the North West), Dunkirk (3630) (located in the Goulburn Valley), and Echuca South (3564) (located in Central VIC). There were no overlapping postcodes for OM and AT. Additionally, there were no postcodes with higher than expected numbers of OM hospitalisations in the North West and Central VIC regions, and no postcodes with higher than expected numbers of AT hospitalisations in the North East region.

### Selected patient characteristics

Table 1 summarises for each of the identified ENT subgroups (i.e. URTI, AT and OM) the number and percent of selected characteristics for Murray PHN patients residing in postcodes with higher than expected hospitalisations compared to those residing in other postcodes in the region. Results show that for OM and URTI, patients from ‘higher than expected’ postcodes were more likely than others to be aged between 0 and 9 years (75.3% vs 62.1%, *p* = 0.01; 60% vs 47.4%, *p* < 0.01 respectively). Conversely, for URTI and AT, patients from ‘higher than expected’ postcodes were more likely than others to be Indigenous (7.5% vs 5.1%, *p* = 0.02; 10.5% vs 5.2%, *p* < 0.01 respectively), require an interpreter (1.8% vs 0.3%, *p* < 0.01; 1.5% vs 0.4%, *p* = 0.01 respectively), and speak a language other than English at home (2.6% vs 1.1%, *p* = 0.01; 2.7% vs 1%, *p* = 0.01 respectively). Additionally for AT, patients from ‘higher than expected’ postcodes were more likely than others to require emergency treatment (99% vs 95.7%, *p* < 0.01). There was no difference between the two patient groups for the remaining combinations of ENT subgroups and patient characteristics.

**Table 1:** Demographic comparison of patients with potentially preventable hospitalisation, ear, nose, and throat conditions in the Murray Primary Health Network region

	*Otitis media*			*Upper respiratory tract infections*			*Acute tonsillitis*		
	**Higher than exp. postcodes, n(%)**	**Other postcodes, n(%)**	*p* value*	**Higher than exp. postcodes, n(%)**	**Other postcodes, n(%)**	*p* value*	**Higher than exp. postcodes, n(%)**	**Other postcodes, n(%)**	*p* value*
*Total*	170	446	n.a	743	1472	n.a	592	1154	n.a
	(100.0)	(100)		(100)	(100)		(100)	(100)	
*Age*			*p* = 0.01			*p* = 0.00			*p* = 0.78
*0–9 year old patients*	128	277		446	698		131	240	
	(75.3)	(62.1)		(60)	(47.4)		(22.1)	(20.8)	
*10–19 year old patients*	11	47		40	85		155	314	
	(6.5)	(10.5)		(5.4)	(5.8)		(26.2)	(27.2)	
*20* + *year old patients*	31	122		257	689		306	600	
	(18.2)	(27.6)		(34.6)	(47.4)		(51.7)	(52)	
*Female patients*	76	206	*p* = 0.74	370	772	*p* = 0.24	343	662	*p* = 0.82
	(44.7)	(46.2)		(49.8)	(52.5)		(58)	(57.4)	
*Indigenous patients*	16	38	*p* = 0.73	56	75	*p* = 0.02	62	60	*p* = 0.00
	(9.4)	(8.5)		(7.5)	(5.1)		(10.5)	(5.2)	
*Patients requiring an interpreter*	0	5	*p* = 0.17	13	4	*p* = 0.00	9	4	*p* = 0.01
	(0)	(1.1)		(1.8)	(0.3)		(1.5)	(0.4)	
*Language other than English patients*	0	8	*p* = 0.08	19	16	*p* = 0.01	16	11	*p* = 0.01
	(0)	(1.8)		(2.6)	(1.1)		(2.7)	(1)	
*Emergency patients*	46	151	*p* = 0.17	688	1385	*p* = 0.18	586	1104	*p* = 0.00
	(27.1)	(33.9)		(92.6)	(94.1)		(99)	(95.7)	
*Public patients*	143	387	*p* = 0.40	667	1285	*p* = 0.09	550	1067	*p* = 0.74
	(84.1)	(86.8)		(89.8)	(87.3)		(92.5)	(92.9)	
*Transfer patients*	5	4	*p* = 0.06	22	49	*p* = 0.64	3	15	*p* = 0.12
	(2.9)	(0.9)		(3)	(3.3)		(0.5)	(1.3)	

^*^Transfer patients refers to patients moving between two different hospitals or hospital campuses where they were assessed or received care and treatment in the first hospital campus; and it is intended that the patient receive admitted care in the second hospital campus

^*^Emergency patients refers to patients who accessed the hospital through the emergency department, as opposed to those who were classified as ‘elective patients’ meaning their admission was pre-planned (for example, a scheduled tonsillectomy)

^*^for Chi-squared statistic; n.a.  *not applicable*

Results from the logistic regression analyses for each of the selected patient characteristics and ENT subgroups are shown in Table 2. The bivariate analyses confirmed the results of the Chi-squared tests above, with the exception of OM patients requiring an interpreter and OM patients who speak a language other than English at home, where unadjusted odds ratio could not be calculated using standard techniques because these characteristics perfectly predicted the likelihood of residing in a postcode with higher than expected hospitalisations due to OM.

**Table 2:** Odds ratios and adjusted odds ratios of patient characteristics

	*Otitis media*		*URTI*		*Acute tonsillitis*	
	**OR** **(95% CI)**	**AOR** **(95% CI)**	**OR** **(95% CI)**	**AOR** **(95% CI)**	**OR** **(95% CI)**	**AOR** **(95% CI)**
*Age*						
*0–9 year old patients*	ref	ref	ref	ref	ref	ref
*10–19 year old patients*	0.51	0.52	0.74	0.73	0.9	0.91
	(0.25–1.01)	(0.26–1.04)	(0.49–1.09)	(0.49–1.09)	(0.68–1.20)	(0.68–1.22)
*20* + *year old patients*	0.55*	0.55*	0.58*	0.58*	0.93	0.95
	(0.35–0.86)	(0.34–0.88)	(0.48–0.70)	(0.48–0.71)	(0.72–1.20)	(0.74–1.24)
*Female patients*	0.94	1.04	0.9	0.98	1.02	1.03
	(0.66–1.34)	(0.72–1.50)	(0.75–1.07)	(0.82–1.18)	(0.84–1.25)	(0.84–1.27)
*Indigenous patients*	1.11	1.02	1.52*	1.45*	2.13*	2.11*
	(0.60–2.06)	(0.54–1.92)	(1.06–2.17)	(1.01–2.08)	(1.47–3.09)	(1.45–3.06)
*Patients requiring an interpreter*	n.c	n.c	6.54*	5.98*	4.44*	2.09
			(2.12–20.11)	(1.33–26.81)	(1.36–14.47)	(0.43–10.22)
*Language other than English patients*	n.c	n.c	2.39*	1.06	2.89*	2.18
			(1.22–4.67)	(0.39–2.86)	(1.33–6.26)	(0.75–6.34)
*Emergency patients*	0.72	0.75	0.79	0.65*	4.42*	3.98*
	(0.49–1.07)	(0.50–1.23)	(0.55–1.11)	(0.45–0.93)	(1.89–10.38)	(1.67–9.45)
*Public patients*	0.81	0.85	1.28	1.13	1.07	0.96
	(0.49–1.32)	(0.51–1.41)	(0.96–1.69)	(0.84–1.51)	(0.73–1.57)	(0.65–1.42)
*Transfer patients*	3.35	4.72*	0.89	0.95	0.39	0.61
	(0.89–12.62)	(1.20–18.54)	(0.53–1.48)	(0.56–1.60)	(0.11–1.34)	(0.17–2.25)

*OR *odds ratio*, AOR *adjusted odds ratio, * p *< 0.05, ref. *reference group*, n.c. *not calculated due to variable perfectly predicting outcome

^*^Transfer patients refers to patients moving between two different hospitals or hospital campuses where they were assessed or received care and treatment in the first hospital campus; and it is intended that the patient receive admitted care in the second hospital campus

^*^Emergency patients refers to patients who accessed the hospital through the emergency department, as opposed to those who were classified as ‘elective patients’ meaning their admission was pre-planned (for example, a scheduled tonsillectomy)

Adjusting for all patient characteristics simultaneously only slightly attenuated the likelihood amongst URTI patients of residing in a postcode with higher than expected hospitalisations if they were Indigenous (OR = 1.52, 95%CI 1.06–2.17 cf. AOR = 1.45, 95%CI 1.01–2.08) or required an interpreter (OR = 6.54, 95%CI 2.12–20.11 cf. AOR = 5.98, 95%CI 1.33–26.81). However, it did reduce the strength of the bivariate association with LOTE patients to non-significance (OR = 2.39, 95%CI 1.22–4.67 cf. AOR = 1.06, 95%CI 0.39–2.86) and increase the strength of the bivariate association with emergency patients to significance (OR = 0.79, 95%CI 0.55–1.11 cf. AOR = 0.65, 95%CI 0.45–0.93), suggesting a degree of confounding with these characteristics in the bivariate analyses.

The picture with AT patients was similar with only a slight attenuation in the likelihood of residing in a postcode with higher than expected hospitalisations if patients were Indigenous (OR = 2.13, 95%CI 1.47–3.09 cf. AOR = 2.11, 95%CI 1.45–3.06) or required emergency treatment (OR = 4.42, 95%CI 1.89–10.38 cf. OR = 5.98, 95%CI 1.33–26.81) but a reduction in the strength of the association to non-significance for patients requiring an interpreter (OR = 4.44, 95%CI 1.36–14.47 cf. AOR = 2.09, 95%CI 0.43–10.22) and LOTE patients (OR = 2.89, 95%CI 1.33–6.26 cf. AOR = 2.18, 95%CI 0.75–6.34), again suggesting a degree of confounding with these characteristics.

For OM patients, the strength of the association between residing in a postcode with higher than expected hospitalisations and being a transfer patient increased to significance (OR = 3.35, 95%CI 0.89–12.62 cf. AOR = 4.72 95%CI 1.20–18.54) after adjusting for all patient characteristics simultaneously, suggesting a degree of confounding with this characteristic in the bivariate analysis. In the univariate analysis, transfer patients were no more likely than others to reside in postcodes with higher than expected numbers of OM presentations. This patient-level attribute only became significant in the multivariate logistic regression for OM suggesting a degree of confounding in the bivariate analysis (we have no plausible explanation for this finding).

For both URTI and OM patients, the strength of the association between residing in a postcode with higher than expected hospitalisations and the 20 + year old age group relative to the 0–9 year old age group did not change after adjusting for all patient characteristics simultaneously (OR = 0.58, 95%CI 0.48–0.70 cf. AOR = 0.58, 95%CI 0.48–0.71 and OR = 0.55, 95%CI 0.35–0.86 cf. AOR = 0.55, 95%CI 0.34–0.88), suggesting no confounding due to age in the bivariate analyses.

## Discussion

### Main findings

While other studies (nationally and internationally) have investigated potentially preventable hospitalisation for other conditions [3–8, 12–14], this is the first study in Australia to report on PPH ENT conditions in the Murray PHN region. The rate of potentially preventable ENT hospitalisations was high for the conditions of upper respiratory tract infections, acute tonsillitis, and otitis media; patients from postcodes with ‘higher than expected’ hospitalisations for these conditions were more likely than others to be aged between 0 and 9 years, Indigenous, or from a culturally and linguistically diverse (CALD) background (patients requiring an interpreter and LOTE patients). These findings may be due to disparities in social determinants and inequalities in access to primary health care for PPH ENT conditions in rural and regional areas. This implication is in line with previous studies where the prevalence of PPH increases with low access to primary health care, and characteristics such as Indigenous identification and CALD population groups [12, 13].

### Place based problems

The higher than expected postcodes identified for URTI, AT, and OM may be due to limited access to primary healthcare in these regions, as the majority of overlapping conditions were in regions outside of major towns. Several studies have reported on the increasing rates of hospitalisation for potentially preventable conditions with increasing distance from metropolitan regions, which may be due to limited primary health care services in outer regions [14, 15]. However, some studies have found that the implementation of rural health clinics in regions lacking primary care services did not significantly reduce hospitalisations for potentially preventable conditions [16]. This may be due to higher rates of multimorbidity experienced by some population groups in remote regions (in particular Medicare beneficiaries and those who identify as Indigenous), and the need for greater infrastructure and staffing to provide more comprehensive services for those with complex needs [16].

### Otitis media and children

Similar to other studies’ findings, being aged between 0 and 9 years was associated with residing in a postcode with higher than expected hospitalisations for OM. OM is one of the most common infectious diseases affecting children, and is responsible for the most commonly performed surgery in children in the management of recurrent acute OM [16, 17]. While not measured in this study, the incidence of OM among Indigenous children has been widely reported on, with the rate of burden from OM in Indigenous children being 8.5 times higher than non-Indigenous children [17]. Despite the greater burden of OM experienced by Indigenous children (and the implications of long term hearing loss), they have lower rates of surgical procedures in the management of acute and recurrent OM [17]. The strongest predictor for hospitalisation of OM in children has been found to be dependent on socio-economic status. Rates of OM hospitalisation indicate social disadvantage to be the greatest predicator [18–20]. Further understanding of the ENT and audiology services available to children in the identified postcodes would provide direction to intervention strategies.

### Culturally and linguistically diverse populations

In this study, requiring an interpreter and speaking a language other than English at home was associated with residing in a postcode with higher than expected hospitalisations for URTI and AT. It has been purported that CALD immigrants arrive in a country in better health than the population of the host country, but their health declines with duration lived in a culturally and linguistically different country [21]. Even in countries such as Canada, where immigrants have the same access as the non-immigrant population to healthcare services (through universal health insurance), they tend to show lower health literacy, difficulty navigating the healthcare system, and understanding how and where to obtain services, or the inability to effectively communicate in the local language [21]. Barriers faced in navigating the primary health care system by the CALD population in regional and rural areas may result in higher hospitalisations rates of PPH conditions, and as identified through this research warrant targeted intervention to reduce PPH of URTI and AT.

## Strengths and limitations

This study is based on all PPH ENT presentations in the Murray PHN region, it can therefore be considered an exhaustive representation. However, there may be inconsistencies within the dataset that are not able to be identified, due to human error when coding conditions in the hospital system, or misinterpretation of the coding. The high rate of ‘unspecified’ ICD codes for each ENT subgroup was not able to be answered within this study; it is most likely an artefact of the coding process where there was not enough information in the medical record for medical coders to apply a more specific code. Subsequent research with the healthcare providers who are entering the codes may explain this limitation. Additionally, it would have been of interest to this research study to analyse the hospital presentations by months to determine the seasonal differences for ENT conditions in the identified postcodes, however this was not possible as all presentations were grouped by years.

The impact of COVID on population hygiene practices, reduced travel, avoidance of emergency departments, and free utilisation of telehealth in primary care should be noted, as there was a significant reduction in PPH ENT presentations in the year of 2020. It can be anticipated that ENT presentations will again increase with the easing of COVID restrictions and reintroduction of primary health care costs.

## Conclusion

This study has identified the leading potentially preventable ENT conditions for hospitalisation in the Murray PHN region, and associated at risk population groups, providing direction to place based interventions. There is an emphasis on the need for place based programs tailored to the population groups identified here, in order to address URTI, AT, and OM ‘hotspots’ in the Murray PHN region. Further investigation of the identified postcodes is warranted to determine access to and utilisation of primary healthcare services in these regions, and the locally available external services to general practice that may fill the gap in ENT identification and management.

### Supplementary Information

**Additional file 1.**

**Additional file 2.**

## Data Availability

The data that support the findings of this study are available from the Victorian Agency of Health Information. However, restrictions apply to the availability of these data, which were used under license for the current study, and therefore are not publicly available. Data are however available from the authors upon reasonable request and with permission of the Victorian Agency of Health Information. The corresponding author of this study Susan O’Neill should be contacted in requesting the studies data (susan.oneill@latrobe.edu.au).
