# Supplementary material for: A five year descriptive analysis of potentially preventable hospitalisations for Ear, Nose, and Throat conditions in regional Victoria, Australia, from 2015 to 2020
Source: BMC Public Health. 2023 Aug 12;23:1536. doi: 10.1186/s12889-023-16448-3 (PMC10422838; doi:10.1186/s12889-023-16448-3)
Supplement: Supplementary file 1 — Additional file 1. [file 12889_2023_16448_MOESM1_ESM.docx]

**Additional file 1. Categorisation of PPH ENT conditions**

| *PPH of ENT conditions* | *ICD-10 codes*  *(Principal diagnosis only)* | *No. of presenting patients 2015-2020* | *Grouped category* |
| --- | --- | --- | --- |
| \| Acute suppurative otitis media \| \| --- \| \| Other chronic suppurative otitis media \| \| Suppurative otitis media unspecified \| \| Otitis media unspecified \| | H66.0  H66.3  H66.4  H66.9 | 27  86  27  516 | Otitis media  *(total: 656)*  *13.6%* |
| \| Streptococcal pharyngitis \| \| \| --- \| --- \| \| Acute pharyngitis dt oth spec organisms \| \| \| Acute pharyngitis unspecified \| \| \| Acute laryngopharyngitis \| \| Other acute URTI of multiple sites \| \| Acute URTI unspecified \| \| Chronic pharyngitis \| | J02.0  J02.8  J02.9  J06.0  J06.8  J06.9  J312 | 44  45  252  9  23  1949  6 | Upper respiratory tract infections (URTIs)  *(total: 2,328)*  *48.3%* |
| \| Streptococcal tonsillitis \| \| --- \| \| Acute tonsillitis dt oth spec organisms \| \| Acute tonsillitis unspecified \| | J03.0  J03.8  J03.9 | 61  99  1672 | Acute tonsillitis  *(total: 1,832)*  *38%* |
